# Clinical Features and Visual Acuity Outcomes in Culture-Positive Endogenous Fungal Endophthalmitis in Southern China

**DOI:** 10.1155/2017/3483497

**Published:** 2017-08-13

**Authors:** Fang Duan, Yao Yang, Zhaohui Yuan, Yongxin Zheng, Zhixing Cheng, Xiaofeng Lin

**Affiliations:** Zhongshan Ophthalmic Center, State Key Laboratory of Ophthalmology, Sun Yat-sen University, Guangzhou 510060, China

## Abstract

**Purpose:**

To report the causative organisms, management strategies, and visual outcomes of culture-proven endogenous fungal endophthalmitis in a case series from southern China.

**Methods:**

We reviewed the microbiological and medical records of patients with culture-positive endogenous fungal endophthalmitis visiting the Zhongshan Ophthalmic Center, Guangzhou, China, between January 1, 2006, and March 31, 2016.

**Results:**

The inclusion criteria were met in 32 eyes of 29 patients. Molds were a common causative organism in 15 patients (51.7%), while yeasts appeared in 14 patients (48.3%). Initial visual acuity (VA) at the level of finger counting or better was significantly related to a good visual outcome (*P* = 0.002). Molds as a causative agent were significantly associated with worse visual outcome than yeasts (*P* = 0.020).

**Conclusion:**

Molds were a common cause of culture-proven fungal endophthalmitis. Endogenous fungal endophthalmitis is generally associated with poor VA outcomes, especially if caused by molds and if the patient's initial VA is too low to permit finger counting.

## 1. Introduction

Endogenous endophthalmitis is an ophthalmic emergency that can have severe sight-threatening complications and generally accounts for 2%–18.5% of all reported endophthalmitis cases [1–6]. Endogenous fungal endophthalmitis is a serious condition with potentially devastating visual outcomes. This condition is usually associated with one or more predisposing systemic conditions, such as diabetes mellitus, liver disease, renal failure, cancer, indwelling catheters, systemic surgery, organ transplantation, acquired immune deficiency syndrome (AIDS), intravenous drug use, and immunosuppressive therapy [7–10]. Occasionally, endogenous fungal endophthalmitis may occur in healthy, immunocompetent patients without any risk factors [11–13].

Fungal infections of endogenous origin can be acquired after systemic infections caused by pathogenic fungi or opportunistic fungi [14]. Many fungi that can cause endogenous fungal endophthalmitis have been reported. Most commonly, endogenous fungal endophthalmitis is associated with *Candida* or *Aspergillus* species in the western countries [7–9] and northern China [10]. However, our previous study reported that the mold was predominant in southern China [6]. The treatments for endogenous fungal endophthalmitis usually include intravitreal antifungal injections, systemic antifungals, and vitrectomy.

The current study aims to report a consecutive series of patients with culture-proven endogenous fungal endophthalmitis treated at a single medical center with specific fungal isolates, treatment strategies, and visual acuity (VA) outcomes.

## 2. Materials and Methods

Microbiological and clinical records were reviewed for all patients undergoing treatment at the Zhongshan Ophthalmic Center, Guangzhou, China, from January 1, 2006, to March 31, 2016, for intraocular culture-proven endogenous fungal endophthalmitis (*n* = 29). This study was performed in compliance with the principles of the Declaration of Helsinki and was approved by the Institutional Ethics Committee of Zhongshan Ophthalmic Center, Sun Yat-sen University.

Intraocular fluid samples were taken from all patients with suspected or diagnosed endogenous fungal endophthalmitis. Fluids from the anterior chamber were aspirated through the limbus using a needle with a 1 mL syringe. Vitreous specimens were obtained through the pars plana prior to intraocular antibiotic injection or vitrectomy. The collected samples were then inoculated onto sheep blood agar and potato glucose agar to be cultured up to 1 week. All the samples were examined daily for detection of fungal growth. Fungi isolates were identified by trained technicians according to the macroscopic characteristics (color and texture) of the colonies. After the list of possible causative organisms had been obtained, the corresponding medical records of patients with positive culture results for fungi were reviewed, and the clinical presentation, treatment strategy, and outcomes were followed and recorded. Treatment and management decisions were made by the ophthalmologist in charge without a predefined study protocol.

The patients were divided into two groups according to whether the final VA reached a favorable visual outcome (at least enough VA to permit finger counting) or not. An independent sample *t*-test, and a chi-square test were used to analyze the difference between the two groups. Fisher's exact test was used if any frequency in the fourfold table was less than five. The level of significance was defined as a *P* value of less than 0.05. All the statistical analyses were performed with SPSS software version 16.0.

## 3. Results

A total of 32 eyes from 29 patients with culture-proven endogenous fungal endophthalmitis were identified in a 10-year period. The average baseline age of these patients was 33.9 ± 16.2 years (range from 1 to 60 years). Thirteen patients (44.8%) were male and 16 (55.2%) were female. The average length of the follow-up period was 3.8 ± 5.2 months (range from 1 week to 2 years). A summary of the clinical characteristics of these patients is provided in Table 1. Bilateral involvement was found in 3 cases (10.3%), and unilateral involvement was found in 26 cases (89.7%).

Eleven patients (37.9%) had a history of surgery in the past 12 months, among which 5 patients (17.2%) received percutaneous nephrolithotomy (PNL) and 6 patients (20.7%) underwent cesarean section, ankle fracture surgery, hernioplasty, childbirth, induced abortion, and hysterectomy. In addition, ten patients (34.5%) had no identifiable infection focus responsible for endogenous endophthalmitis.

All 3 cases with bilateral disease were identified to have positive intraocular culture results in at least one eye. The presence of mold (15 patients, 51.7%) was slightly higher than that of yeasts (14 patients, 48.3%). The 15 mold cultures included *Aspergillus* (*n* = 7), *Fusarium* (*n* = 2), *Mucor* (*n* = 2), *Helminthosporium* (*n* = 1), and *Penicillium* (*n* = 1); 2 molds could not be identified. Among the 14 yeast cultures, only 3 were identified as *Candida albicans.*

Among all 29 patients (32 eyes), 26 patients (28 eyes) underwent pars plana vitrectomy (PPV), and 19 patients (21 eyes) were tamponaded with silicone oil subsequently. Three patients could not receive PPV due to severe opacity and edema of the cornea. One patient with bilateral endophthalmitis did not receive surgery in the better eye because the VA had improved after treatment with antifungal medicine.

As shown in Table 2, 30 eyes (27 patients) with initial and final VAs were available for the analysis of possible prognostic factors for the final visual outcome. Two subjects were excluded because they were too young to conduct a VA test. Patients with different levels of initial VA and species of fungi showed significant differences between poor (worse than FC) and relatively good prognosis (FC and better) (*P* = 0.002 and *P* = 0.020, resp.). Whether the patient underwent surgical treatment and whether the tamponading agent (silicone oil) was used did not affect the final visual outcome (*P* = 0.563 and *P* = 0.225, resp.).

## 4. Discussion

Endogenous fungal endophthalmitis is a rare intraocular infection resulting from the systemic hematogenous spread of the organism to the eye. In the present study, the medical records and visual outcome of 29 subjects with endogenous endophthalmitis were reviewed. We revealed that a majority of patients had a positive history of surgery, and nearly 1/3 of the cases of endogenous endophthalmitis occurred in otherwise apparently healthy immunocompetent individuals without any infection focus elsewhere in the body. The presence of mold was slightly higher than that of yeasts. The risk factors affecting the visual outcome of endogenous fungal endophthalmitis included initial VA worse than FC and type of fungus (mold).

In previous studies, systemic diseases including diabetes mellitus and renal disease were the main risk factors of endogenous endophthalmitis [8, 15, 16]; however, in our case series, the major predisposing risk factor was recent history of surgery. There were several reasons that may account for this phenomenon. First, the study site is an ophthalmology-specialized hospital; most patients with severe systemic diseases were more likely to visit general hospitals looking for care from both ophthalmologists and other physicians. Second, the abuse of antibiotics in China [17], especially for postoperative patients, may increase the risk of endogenous fungal infection.

Though in most of the cases of endogenous endophthalmitis one or more risk factors are identified, a few cases among otherwise healthy and immunocompetent individuals have also been reported [11–13]. The etiology and pathogenesis are not fully identified. The visual outcome of such patients is usually poor [13]. The findings in the 10 cases of this study were in agreement with the previous reports. The major organism accounting for these cases was *Aspergillus*. The mononuclear phagocyte defenses are usually the first line of defense against *Aspergillus* infections, followed by the neutrophils [18, 19]. Some deficiency may exist in the mononuclear phagocytes and neutrophils defenses of these patients. However, we did not have records of immunity tests in these patients. A detailed examination of immune function should be conducted for such cases to explore the underlying causes in future work.

Most commonly, yeast was the main cause of endogenous fungal endophthalmitis in western countries [7–9, 20, 21], and most of them were due to *Candida*. A study in northern China had similar results [10]. However, in our study, the prevalence of mold was more than that of yeast species. Additionally, a lower percentage of *Candida* was found in our endogenous fungal endophthalmitis cases. Environmental and geographic factors may have contributed to this discrepancy because the spectrum and virulence of causative organisms vary depending on the region and the environment. On the other hand, the patients already had severe endophthalmitis, which is more likely to be associated with mold infection, before they were referred to our institution.

PPV was an important management strategy for patients with endophthalmitis and a surgical method to obtain vitreous samples for diagnosis. An early diagnosis can be made, and interventions including vitrectomy can be carried out to improve the visual outcome [22]. Vitrectomy can reduce the burden of microorganisms and inflammatory mediators present in the vitreous cavity. Lee et al. reported that early vitrectomy in patients with initially good visual presentation was significantly related to a successful visual outcome [23]. In this case series study, 70.8% (17/24) patients who had improved VA outcome underwent simple or combined PPV. This again emphasizes the role of vitrectomy in treating endophthalmitis, even for cases caused by endogenous fungal growth. However, not all patients who underwent PPV showed improved VA after surgery, and chi-square analysis indicated that PPV did not influence the visual outcome significantly. A possible reason may be the late presentation of these patients, implying that early interventions were not made. The small sample size can also produce misleading results. Though PPV may not improve VA in an obvious manner, it is necessary for cases with mass vitreous opacity and rapid progression [24].

Previous studies had found that good initial visual acuity greater than FC was related to a good visual outcome [23, 25]. This is consistent with our analysis, which suggests that good initial VA is an important predictor for visual outcome. Mold endophthalmitis, however, showed worse visual prognosis than yeast cases. This finding agrees with other studies [10, 20], and the possible mechanism may be the invasive patterns of *Aspergillus* infection in the retina and choroid [26]. These findings indicate that earlier and more aggressive interventions should be made for those with mold-induced endophthalmitis to maintain visual acuity as much as possible.

The limitations of the study should be mentioned. This study was retrospectively designed and lacked universal criteria for diagnosis and treatments. The youngest subjects in our series were 1 and 2 years old, limiting the full assessment of visual outcome. Detailed categories of the fungus species were also absent in some cases. Finally, the small sample size may produce bias in the analysis and conclusion.

In conclusion, we assessed the clinical features and visual outcome of 29 patients with culture-proven endogenous fungal endophthalmitis. The major predisposing risk factor was recent history of surgery. The main organism in patients without identifiable systemic risk factors was *Aspergillus*. The presence of mold was slightly higher than that of yeasts. The factors affecting visual outcome of endogenous fungal endophthalmitis included initial VA and species of fungus. Whether PPV with silicone oil tamponade was used or not may not influence the visual outcome in severe cases.

## Figures and Tables

**Table 1:** Clinical summary of patients with endogenous fungal endophthalmitis.

Patients	Age	Gender	Eye	Fungus	Predisposing risk factors	Initial vision	Vitrectomy	Silicone oil	Final vision
1	57	M	OD	*Aspergillus terreus*	IT, LD	LP	Yes	Yes	FC
2	2	F	OS	Mold	None	—	Yes	Yes	—
3	43	F	OS	*Mucor*	PNL	FC	Yes	No	20/63
4	41	F	OD	Yeast	PNL	FC	Yes	Yes	20/50
5	60	M	OD	Yeast	Cholecystitis Hernioplasty	FC	Yes	No	20/800
6	35	F	OD	Yeast	Cervicitis	FC	Yes	No	20/800
7	30	F	OS	Yeast	Hysterectomy	HM	Yes	Yes	HM
8	28	M	OU	Yeast	AFS	20/50 20/400	No Yes	No No	20/40 20/25
9	10	F	OS	*Helminthosporium*	None	HM	Yes	Yes	FC
10	55	M	OS	*Aspergillus nidulans*	TB	HM	No	No	HM
11	30	F	OS	*Aspergillus nidulans*	None	0.1	Yes	No	NLP
12	43	F	OD	Yeast fungus	None	HM	Yes	Yes	LP
13	53	M	OD	*Candida albicans*	PNL	FC	Yes	Yes	FC
14	13	M	OD	*Fusarium*	None	LP	No	No	NLP
15	6	M	OD	*Aspergillus*	None	20/200	Yes	Yes	20/400
16	51	M	OU	*Candida albicans*	PNL	20/200 FC	Yes Yes	Yes Yes	20/32 20/40
17	26	M	OS	*Aspergillus ochraceus*	None	LP	Yes	No	HM
18	44	M	OD	Yeast	Pneumonia	FC	Yes	Yes	20/800
19	39	M	OD	*Aspergillus flavus*	None	HM	Yes	Yes	20/400
20	34	M	OS	*Penicillium*	LD	LP	Yes	No	NLP
21	27	F	OD	Yeast	IA	FC	Yes	Yes	20/63
22	50	F	OU	*Candida albicans*	PNL	FC HM	Yes Yes	Yes Yes	20/200 20/400
23	26	F	OD	*Mucor*	CM	LP	Yes	Yes	HM
24	34	F	OS	Yeast	CS	FC	Yes	Yes	20/100
25	1	F	OS	*Fusarium moniliforme*	None	—	Yes	Yes	—
26	25	F	OS	Yeast	Pneumonia	FC	Yes	Yes	20/800
27	25	F	OS	Yeast	Childbirth	FC	Yes	Yes	20/400
28	56	F	OS	Mold	None	HM	Yes	Yes	HM
29	31	M	OD	*Aspergillus flavus*	IT	HM	No	No	20/200

*Note*. NLP: no light perception; HM: hand motion; FC: finger counting; OD: right eye; OS: left eye; OU: both eyes; IT: immunosuppressive therapy; LD: liver disease; PNL: percutaneous nephrolithotomy; CS: cesarean section; AFS: ankle fracture surgery; TB: tuberculosis; IA: induced abortion; CM: colpitis mycotica.

**Table 2:** Prognostic factors associated with favorable visual outcome.

Factors	Final visual outcome		*P* value
	FC or better than FC	Worse than FC	
Initial visual acuity			
FC or better than FC	16	1	0.002
Worse than FC	5	8	
Vitrectomy			
No	2	2	0.563
Yes	19	7	
Silicone oil			
No	6	5	0.225
Yes	15	4	
Fungi			
Yeast	15	2	0.020
Mold	6	7	
